# Influence of dental metallic artifact from multislice CT in the assessment of simulated mandibular lesions

**DOI:** 10.1590/S1678-77572010000200009

**Published:** 2010

**Authors:** Andréia PERRELLA, Patricia de Medeiros Loureiro LOPES, Rodney Garcia ROCHA, Marlene FENYO-PEREIRA, Marcelo de Gusmão Paraíso CAVALCANTI

**Affiliations:** 1 DDS, MSc, Department of Stomatology, Dental School, University of São Paulo, São Paulo, SP, Brazil.; 2 DDS, PhD, University Center of João Pessoa, João Pessoa, PB, Brazil.; 3 DDS, MSc, PhD, Department of Stomatology, Dental School, University of São Paulo, São Paulo, SP, Brazil.

**Keywords:** Mandible, Tomography, X-ray computed, Pathology

## Abstract

**Objective:**

This study evaluated the influence of metallic dental artifacts on the accuracy of simulated mandibular lesion detection by using multislice technology.

**Material and Methods:**

Fifteen macerated mandibles were used. Perforations were done simulating bone lesions and the mandibles were subjected to axial 16 rows multislice CT images using 0.5 mm of slice thickness with 0.3 mm interval of reconstruction. Metallic dental restorations were done and the mandibles were subjected again to CT in the same protocol. The images were analyzed to detect simulated lesions in the mandibles, verifying the loci number and if there was any cortical perforation exposing medullar bone. The analysis was performed by two independent examiners using e-film software.

**Results:**

The samples without artifacts presented better results compared to the gold standard (dried mandible with perforations). In the samples without artifacts, all cortical perforation were identified and 46 loci were detected (of 51) in loci number analysis. Among the samples with artifacts, 12 lesions out of 14 were recognized regarding medullar invasion, and 40 out of 51 concerning loci number. The sensitivity in samples without artifacts was 90% and 100% regarding loci number and medullar invasion, respectively. In samples with artifacts, these values dropped to 78% and 86%, respectively. The presence of metallic restorations affected the sensitivity values of the method, but the difference was not significant (p>0.05).

**Conclusion:**

Although there were differences in the results of samples with and without artifacts, the presence of metallic restoration did not lead to misinterpretation of the final diagnosis. However, the validity of multislice CT imaging in this study was established for detection of simulated mandibular bone lesions.

## INTRODUCTION

Several studies have demonstrated the application of computed tomography (CT) in the diagnosis and treatment planning of lesions of the jaws^1-3^. CT images provide important information about cortical margins, extent of lesion, and involvement of surrounding structures, as well as knowledge of the cortical margin^1-3^. However, the examinations can be disturbed by serious artifacts caused by metallic fillings in teeth^2,6,16,18,19,25,27,30^. Many authors have already used simulated bone lesions in the jaws to compare conventional radiographic techniques^5,17,22^, and also more recently in singleslice CT^20,21,24^ and cone beam computed tomography (CBCT)^23^.

High atomic numbers of contrast agents or metal implants result in increased fraction of photoelectric interactions causing photopenic holes in the projection datathat are displayed on CT images as sunburst streaks^11,18^, which emanate radially from the site of the metal object^14^. The severity of “sunburst” artifact was found to be related to the physical size of the fixation hardware and its composition^11-14^. The reduction of metal artifacts in x-ray CT has important clinical applications, and many authors are searching for a way to reduce them^14,18,28^.

The recent development of multislice CT promises to reduce hardware artifacts among other advantage as unprecedented speed, the capability to cover large volumes, isotropic imaging, soft-tissue imaging, and ease of image interpretation^7,8,10,11^. The aim of this study was to evaluate the influence of metallic dental artifacts on the accuracy of simulated mandibular lesion detection by using multislice technology

## MATERIAL AND METHODS

**Figure 1:**
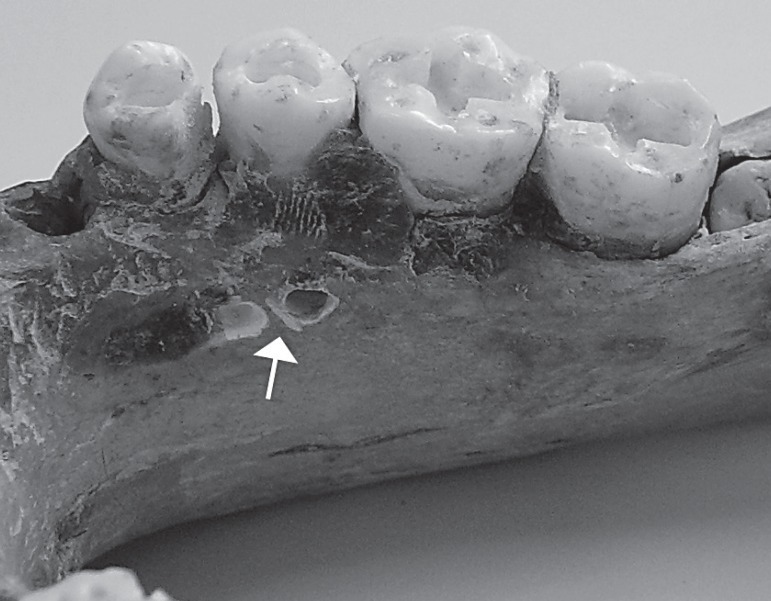
Right side of a dry mandibular body. The arrow shows lingual cortical and medullar simulated perforations

Fifteen dry mandibles were examined in which lesions involving only cortical or cortical and medullar bone were produced with a #1012 round bur (diameter of active point: 1mm) mounted in a high-speed handpiece. These lesions were located in the buccal or lingual cortex of mandibular body and symphysis region with different dimensions, shapes and loci number. In some cases, the bur just touched the cortical bone, and, in others it was inserted into medullar bone (Figure 1). Pendular movements were carried out to get larger simulated lesions, therefore the diameter of the simulated lesions ranged from 1 mm to 3 mm and the depth ranged from 0.5 mm to 3.0 mm. A total of 51 perforations were done. In 7 mandibles, the perforations were unilocular, located in lingual body of the mandible and, in all of them there was cortical perforation exposing medullar bone. In 8 mandibles, the simulated lesions were multilocular (ranging from 3 loci to 9 loci), located in lingual body of the mandible; in 2 of them there were perforations in the buccal cortex of the body. From the 15 mandibles, only 1 did not have cortical perforation exposing medullar bone.

Subsequently, the mandibles were imaged on 16 rows multislice CT (Aquilion, Toshiba Medical Inc., Tustin, CA, USA), using the following parameters: 0.5 mm of slice thickness, with 0.3 mm interval of reconstruction with time of 0.5 s, (120 KVp, 300mA, and matrix 512 x 512) using a bone tissue filter. Scan angle and field of view were kept constant. The mandibles were put into a plastic bucket, completely covered with water (in order to attenuate the radiation, resembling soft tissue), and fixed at the same position as it is proceeded in vivo, using cotton sheets to support them. The specimens were scanned from its base to the condyle region by axial sections. The scanning plane was positioned parallel to mandibular base. The Gantry angulations varied according to mandible base angulations. Amalgam restorations, metallic crowns and metallic fixed prostheses (3 elements) were made in teeth of the mandibles and they were scanned again with the same parameters, at the same position. Each mandible presented at least 2 teeth with some metallic component. Both sides of the mandible presented metallic restorations. The amalgam restorations were done directly in the mandibular teeth, the crowns and prostheses were fixed to the teeth or to the bone crest with utility wax. All procedures were conducted after approval by the local Research Ethics Committee.

**Figure 2:**
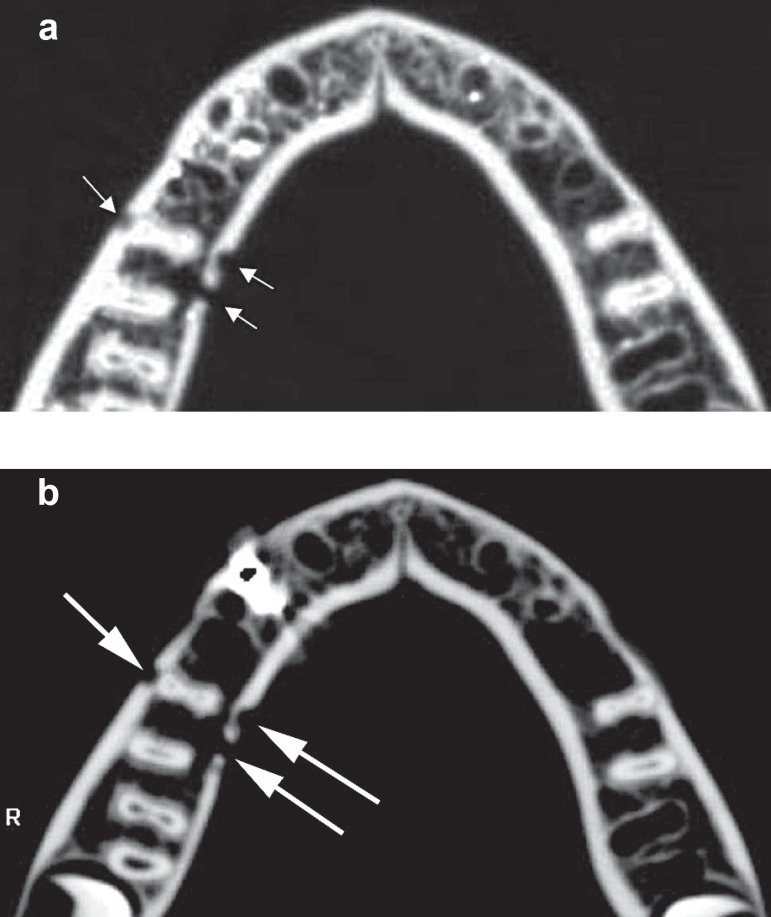
a) Axial CT of the mandible without metallic restorations and prostheses. The arrows indicate one cortical perforation in the buccal side and two perforations in the lingual side (right side). The more distal simulated lesion has also medullar invasion. b) Axial CT of the mandible with metallic restorations and prostheses. Even with the presence of the metallic artifact, there was no misinterpretation regarding the loci number and medullar invasion (arrows) compared to the imaging findings of figure 2a

The data were sent in DICOM (Digital Imaging Communication in Medicine) format to a workstation, recorded in a CD-ROM-R, and transferred to an independent computer (Pentium 4, 60 GB HDD, 512Mb RAM, running Windows XP). All images were displayed and analyzed using commercially available software eFilm (1.5.3 version, Merge Healthcare, eFilm, Milwaukee, WI, USA), and interpreted independently by two experienced examiners (oral and maxillofacial radiologists). The analyses of the images were performed in a random order of the protocols, in different sessions. The examiners were told that perforations were done in mandibular body, but they were totally blinded about the aspects of the lesions in each mandible. The simulated lesions were performed by a third observer who was unaware of any information about the evaluation process. The examiners were asked to judge whether they correctly identified different conditions as if there was cortical perforation and the loci number of each present lesion in both protocols: with and without artifacts (Figures 2a and 2b).

The statistical analysis was carried out using validity test and Kappa value. The validity test is represented by Youden´s J index, which is one way to attempt summarizing test accuracy into a single numeric value (sensitivity + specificity – 100).

## RESULTS

The data from the image analysis are shown in Tables 1-3. The samples without artifacts presented better results compared to the gold standard (dried mandibles with perforations) (Tables 1 and 2): for medullar invasion all lesions were identified (Table 1) and for loci number analysis 46 loci out of 51 were detected (Table 2).

**Table 1:** Distribution of the results (absolute values) of the CT image with and without artifacts compared to the gold standard in the analysis of cortical perforation

	**With artifacts**	**Without artifacts**	**Gold Standard**
True positives	12	14	14
False Negatives	2	0	-
True Negatives	1	1	1
False Positives	0	0	-

**Table 2:** Distribution of the results (absolute values) of the CT image with and without artifacts compared to the gold standard in the analysis of loci number detection

	**With artifacts**	**Without artifacts**	**Gold Standard**
True positives	40	46	51
False Negatives	11	5	-
True Negatives	0	0	0
False Positives	0	0	-

**Table 3:** Sensitivity and specificity for loci number detection and cortical perforation in all protocols

	**samples without artifacts**		**samples with artifacts**	
	**Loci number detection**	**Cortical Perforation**	**Loci number detection**	**Cortical Perforation**
Sensitivity	90%	100%	78%	86%
Specificity	100%	100%	100%	100%
Validity	90%	100%	78%	86%

The sensitivity, specificity and validity are presented in Table 3. In evaluation of loci number, the sensitivity was 90% without artifacts and 78% with artifacts. The value of specificity was 100%. Regarding medullar invasion, there was 100% of sensitivity without artifacts, and 86% with artifacts interference. The specificity was 100%. The results of the validity test represented by Youden’s J index were 90% and 78% for loci number and 100% and 86% for medullar invasion, in samples without and with metallic artifact, respectively. Using chi-square test for comparison of samples with and without artifacts, no significant differences were found for loci number (p=0.068) and or medullar invasion (p=0.207).

Kappa statistics was used as a way to quantify the level of agreement between the examiners in order to test the reproducibility of the methodology. The Kappa value ranged from -1 (lower level of agreement) to 1 (higher level of agreement). The Kappa value obtained for all analysis performed in this study was 1.

## DISCUSSION

Many authors have discussed the importance of acquisition parameters as slice thickness in bone lesion evaluation^4,9,26^. Shaha^26^ stated that for detailed evaluation of the mandible is essential to obtain the CT scans with bone windows and narrow cuts, since the accuracy found in his work was 68%, using a singleslice CT. According to Baxter and Sorenson^4^, the number of lesions is inaccurate when the diameter is comparable to or less than the CT slice thickness^12^. Furthermore, Cavalcanti, et al.^9^ demonstrated a high false positive and false negative rates when determining bone invasion in mandible, because 3-mm-thick axial slices are used, and also most authors suggested that thin slices are needed to detect more bone details^4,9,26^.

Multislice technique makes possible to obtain thinner slices, with fast scanning time, allowing the capability to cover large volumes, isotropic imaging, reduced hardware artifacts, and improvement of image quality detailed^7,10-11^. In the present work using 0.5 mm slice thickness with a thinner interval of reconstruction (0.3 mm), 90% of sensitivity and 100% of specificity were found regarding the number of simulated lesions. Regarding the medullar invasion of these lesions, the sensitivity and specificity was 100%. It may be infered that these values are high since in some cases the loci size simulated were very small (the depth was greater than the bur diameter of 1 mm). It may be speculated that for this size of lesions, thinner cuts obtained with CT multislice technology allowed expressive results, as demonstrated in Table 1 since wefound a 90% sensitivity value.

Huntley, et al.^15^ (1996) found a sensitivity of 62.5% in analysis of bone invasion by squamous cell carcinoma using CT parameters of 1.5 mm slice thickness with 1.5 mm of interval of reconstruction, and stated that these results could be improved by reducing slice thickness. There was a considerable difference between the sensitivity values obtained by Huntley, et al.^15^ (1996) and that of the present study, even if we consider the simulated lesions differs from pathologic ones. In our research, the reason for this high sensitivity value (90% without dental metallic artifact) for loci number can be the reduction of the CT parameters (0.5mm of slice thickness and 0.3 mm of interval of reconstruction). This sensitivity can be considered also high since the lesions size, in some cases, were smaller than 1mm, differing from Huntley’s study, which lesions were in alveolar crest spreading into lingual cortex.

It is demonstrated that metallic filling in teeth can cause serious artifacts in tomographic images that can lead to misinterpretation of lesions in craniofacial structures as the mandible^12-13,26-28^. Shaha^26^ found that CT scanning was not very helpful in detecting mandibular invasion of carcinoma of the floor of mouth because of the presence of irregular dental sockets and artifacts. The examinations are seriously disturbed by fillings on teeth^27^. On the other hand, some authors have already suggested that multislice technology can reduces these artifacts, so they suggests the use of multislice technology in patients with metallic hardware in their bodies^7,10-11^, corroborating to our purpose that was to test whether or not the artifact could influence in detection of the lesions. Although the present study showed better results in sensitivity of multislice CT without artifacts for loci number identification and detection of cortical perforation (90% and 100%) than in samples with artifacts (78% and 86%) respectively, there were no significant difference between these results (p>0.05). Furthermore, the literature showed that metallic artifact could influence negatively the interpretation of images in CT when singleslice technology was used^12-14,26,28^.

Although this study used mechanical pseudolesions that are not radiographically identical to those developed naturally, simulated lesions were chosen in order to have a defined pattern to compare samples with and without artifacts. Simulated bone lesions have been widely used to compare radiological techniques for bone observation^5,17,20,21,22,24,29^, and are already in use to test the accuracy of CBCT protocols ^23^. Pinsky, et al.^23^ (2006) used simulated lesions to test linear measurements in CBCT, but they made 4-8-mm defects, which are larger than those prepared in the present work. Although the lesions were arbitrarily disposed, their shapes and localization were known. In such experiments, water was added to produce an environment closer to bone *in vivo*, while in the present experiment, water was not used into the bucket in order to achieve a position as close as possible of the position for mandibular examination *in vivo*, in such a way that the x-ray was only attenuated by the plastic bucket and the cotton sheets that supported them. Therefore, our *in vitro* validation study aimed at establishing whether multislice CT 16 rows are accuratefor determining osseous defect sizes in the presence or absence of metallic artifacts, and demonstrates that clinically acceptableaccuracy can probably be obtained for mandibular applications requiring evaluation of small osseous defects.

It is the authors` opinion that multislice CT technology may improve the results of early detection of bone lesions *in vivo*, as good sensitivity and sensibility rates were obtained even with tiny simulated lesions. Furthermore, Kappa values of 1 were obtained for all evaluations, which suggest that the methodology is not examiner dependent. Since image quality on patients is decreased by the presence of soft tissue and possible patient movement during scanning^23^,further studies are needed to confirm the present results clinically.

## CONCLUSION

Although, there were differences in the results of samples with and without artifacts, the presence of metallic restoration did not lead to misinterpretation of the final diagnosis. However, the validity of multislice CT imaging in this study was established for detection of simulated mandibular bone lesions.
